# A molecular map of mesenchymal tumors

**DOI:** 10.1186/gb-2005-6-9-r76

**Published:** 2005-08-26

**Authors:** Stephen R Henderson, David Guiliano, Nadege Presneau, Sean McLean, Richard Frow, Sonja Vujovic, John Anderson, Neil Sebire, Jeremy Whelan, Nick Athanasou, Adrienne M Flanagan, Chris Boshoff

**Affiliations:** 1 Cancer Research UK, Viral Oncology Group, Wolfson Institute for Biomedical Research, Gower Street, University College London, London, WC1E 6BT, UK; 2 Division of Cell and Molecular Biology, Biochemistry Building, Faculty of Life Sciences, Imperial College London, South Kensington Campus, London, SW7 2AZ, UK; 3 Institute of Orthopaedics and Department of Pathology, Royal National Orthopaedic Hospital, Stanmore, Middlesex, HA7 4LP, UK; 4 Unit of Molecular Haematology and Cancer Biology, Institute of Child Health and Great Ormond Street Hospital, Guildford Street, London, WC1N 1EH, UK; 5 Department of Pathology, Great Ormond Street Hospital for Children, London, WC1N 3JH, UK; 6 London Bone and Soft Tissue Tumour Service, University College London Hospitals, London, UK; 7 Department of Pathology, Nuffield Department of Orthopaedic Surgery, Nuffield Orthopaedic Centre, Headington, Oxford, OX3 7LD, UK

## Abstract

**Background:**

Bone and soft tissue tumors represent a diverse group of neoplasms thought to derive from cells of the mesenchyme or neural crest. Histological diagnosis is challenging due to the poor or heterogenous differentiation of many tumors, resulting in uncertainty over prognosis and appropriate therapy.

**Results:**

We have undertaken a broad and comprehensive study of the gene expression profile of 96 tumors with representatives of all mesenchymal tissues, including several problem diagnostic groups. Using machine learning methods adapted to this problem we identify molecular fingerprints for most tumors, which are pathognomonic (decisive) and biologically revealing.

**Conclusion:**

We demonstrate the utility of gene expression profiles and machine learning for a complex clinical problem, and identify putative origins for certain mesenchymal tumors.

## Abstract 2

A comprehensive study of the gene expression profile of 96 mesenchymal tumors identifies molecular fingerprints for most tumors in this group.

## Background

Tumors of bone and soft tissue are a wide spectrum of benign and malignant neoplasms (sarcoma) derived from mesenchymal precursor cells (hereafter referred to as mesenchymal tumors) [1,2]. Many show heterogeneous patterns of differentiation or exhibit little similarity to differentiated mesenchymal tissues, while others have diverse cellular morphology (pleomorphism). Thus, specialist expertise is required for diagnosis as the histopathology of mesenchymal tumors is often overlapping or indistinct. With the introduction of neo-adjuvant cytotoxic therapies, diagnosis has become even more challenging as pathologists must rely increasingly upon needle core biopsies that produce only small quantities of tissue for immunohistochemistry and histopathological diagnosis. Furthermore, molecular therapies have been developed targeting oncogenic pathways that may transcend the current histopathological categories.

The discovery of definitive oncogenic gene fusions for certain mesenchymal tumors has aided pathologists greatly. These include the EWS-ERG or EWS-FLI1 fusion transcripts for Ewing's sarcoma (EWS) [3-5], or the SYT-SSX fusion transcript for synovial sarcoma [6,7]. Also, reverse transcriptase polymerase chain reaction (RT-PCR) has become the gold standard for diagnosis. These 'simple sarcomas' are ideal candidates for targeted therapy, with relatively stable karyotypes and stereotypical molecular pathology [8]. Nonetheless, chromosomal translocations are confirmatory for only a fraction of mesenchymal tumors while those with complex karyotypes remain diagnostically challenging.

There are many gene expression microarray (GEM) studies covering a range of mesenchymal tumors [9-28]. These studies have proved the general applicability of GEM for the diagnosis of mesenchymal tumors. Yet each study compares a fraction of the mesenchymal tumors in isolation. They do not address the spectra of disease nor the challenges of diagnostic pathology, which deals with many confounding diagnoses. Here we present a more comprehensive study encompassing representative tumors derived from all mesenchymal tissue types, as well as more poorly differentiated tumors.

Using a machine learning model, we assess the overall utility of GEM for the diagnosis of 19 types of mesenchymal tumors. We find both expected and unexpected relationships between certain mesenchymal tumors, and ascertain expression fingerprints for decisive diagnostic or pathognomonic features for most tumor classes. These fingerprints give clues to the etiology of several mesenchymal tumors.

Our machine learning model fits the data in two steps. The first step incorporates biological assumptions by merging related tumors into broader groups. Step two splits the broader groups into specific tumors. This latter process is derived by expert decision, rather than automated model selection. We consciously mirror the clinical diagnostic method of progressively resolving general differentiation types into specific tumors in a branch-wise (or tree-like) method. The use of a decision process structured by prior knowledge (together with a well-suited feature selection algorithm) gives our model an estimated error of about 0.1. Although further study and validation will be required to bring it to clinical use, we believe this method is a prototype for the extension of GEM and machine learning to other complex diagnostic problems. The GEM data from this study are available from the European Bioinformatics Institute (EBI) public repository ArrayExpress (accession no. E-MEXP-353) [29].

## Results

**Figure 1:**
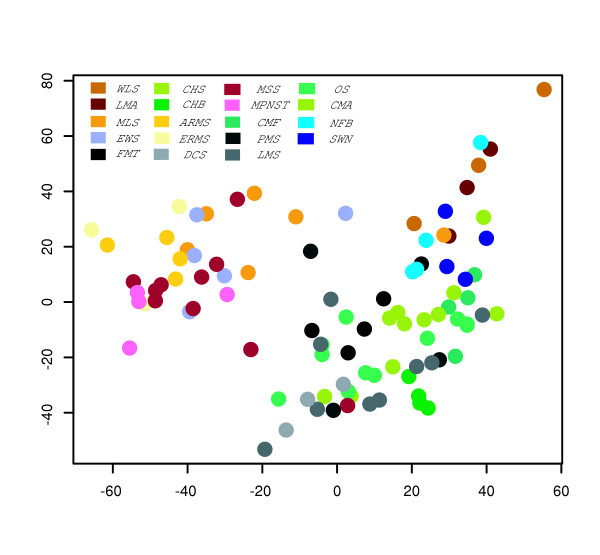
Multi-dimensional scaling of all 96 mesenchymal tumor samples. There are 19 types of tumor shown; the color coding of which is used consistently for all figures. All gene expression values were used to calculate the inter-sample Euclidean distance matrix. The distances are translated here onto a two-dimensional plane using the classical cmd-scale algorithm of R. The stress of the plot was 0.34; an index of the goodness of fit between the original distance matrix and the MDS distance (see Materials and methods). WLS, well-differentiated liposarcoma; LMA, lipoma; MLS, myxoid liposarcoma; EWS, Ewing's sarcoma; FMT, desmoid fibromatosis; CHS, chondrosarcoma; CHB, chondroblastoma; ARMS, alveolar rhabdomyosarcoma; ERMS, embryonal rhabdomyosarcoma; DCS, de-differentiated chondrosarcoma; MSS, monophasic synovial sarcoma; MPNST, malignant peripheral nerve sheath tumors; CMF, chondromyxoid fibroma; PMS, pleomorphic sarcoma; LMS, leiomyosarcoma; OS, osteosarcoma; CMA, chordoma; NFB, neurofibroma; SWN, schwannoma.

We determined the gene expression profile of 96 mesenchymal tumors, representing 19 different sub-types, using the Affymetrix HG-U133Av2 oligonucleotide GeneChip^®^. A multi-dimensional scaling (MDS) of tumor samples using all genes reveals much structure in the data (Figure 1). We chose MDS rather than hierarchical clustering for a more compact and comprehensible summary. An average linkage hierarchical clustering using the same distance matrix is given in Additional data file 1 online.

The differentiated tumors largely cluster in groups reflecting common tissue types. Examples are the neurofibroma (NFB) and schwannoma (SWN), the alveolar (ARMS) and embryonal rhabdomyosarcoma (ERMS), and the well-differentiated liposarcoma (WLS) and lipoma (LMA). Similarities in the GEM profiles of malignant peripheral nerve sheath tumors (MPNST) and monophasic synovial sarcoma (MSS) were previously reported and this relationship is supported in our data [18]. Likewise, the relation between the small round blue cell tumors: EWS, ARMS and ERMS is clear from this plot. An interesting finding was the similarity of the chordoma (CMA), a rare tumor arising along the midline to the chondrosarcoma (CHS). Reflecting the experience of histopathological diagnosis, the osteosarcoma (OS), pleomorphic sarcoma (PMS) and leiomyosarcoma (LMS) show the greatest diversity being dispersed throughout the other samples.

The MDS is based on distances calculated from the whole molecular signature (all genes or probesets) (Figure 1). Thus, confounding factors such as intercalating tissue, tumor site or patient age and sex may affect the resultant pattern.

### Supervised learning

The key to the success of supervised learning is to focus on a small set of strongly distinguishing features, thus ignoring confounding factors. Strong feature selection is appropriate in this study as we have sound histological evidence that there are underlying patterns to be uncovered (at least for most tumors). This reduced set of features (or genes) gives a molecular fingerprint of the tumors that is both diagnostic and descriptive. Our feature selection method is suited to this application as it selects ten genes evenly for each class. Using a simple k = nearest neighbors (k-NN) machine learning algorithm and the extended feature selection algorithm we attempted initially to create a model for all tumors simultaneously (see Materials and methods section for more detail on feature selection and k-NN algorithm). The overall cross-validation error was 0.33 compared with a random guessing error of 0.96 +/- S.E. 0.01 (see Materials and methods: classification algorithm). Therefore, even with this simplest approach, the machine learning model is fairly successful.

The true error, and thus the generalization to other datasets, is likely to be underestimated (see Discussion). However, an important control shows that our method does not over-fit the data excessively. In simple terms, the method does not erroneously fit this particular dataset using random patterns. After randomly permuting all the labels to create a semi-random dataset we tried to cross-validate these false data (see Materials and methods: classification algorithm). The error was 0.94 +/- S.E. 0.01 (n = 10 permutations) so was therefore not significantly better than random guessing (Table 1).

### Two-step model

The majority of errors from the simple model above were as expected between the adipocytic tumors LMA, WLS and myxoid liposarcoma (MLS), between MPNST and MSS, and between LMS, PMS and de-differentiated chondrosarcoma (DCS). By considering unsupervised learning methods (MDS and hierarchical clustering) plus biological assumptions and trial-and-error, we arrived at an improved two-step method. This was designed, in part, to mirror the thought processes involved during clinical histopathological diagnosis. This two-step model is, thus, expert derived and not an automated procedure.

**Figure 2:**
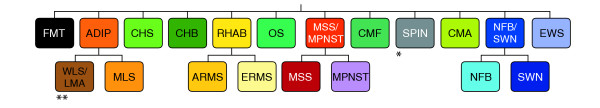
Schematic of two-step model. In order to successfully classify the sarcoma with the minimum of errors a two-step approach was used. A mixture of single sarcoma and composite classes were used for prediction in step one. Then, in step two, composite classes were separated into their constituent tumors. * The SPIN (spindle-like) group comprising PMS, LMS, and DCS could not be separated by our model. ** The WLS and LMA could not be separated by our model but were distinct from MLS. ADIP, adipocytic tumors; RHAB, rhabdomyosarcoma.

In step one, we grouped tumors into composite classes assumed to reflect similar pathways or levels of differentiation; MPNST/MSS, PMS/LMS/DCS, ARMS/ERMS, NFB/SWN and LMA/WLS/MLS. In step two, we decomposed the composite classes into their sub-types. This decision process is illustrated in color code in Figure 2.

Using the same feature selection and learning algorithm as the initial method, the new model is remarkably effective in the first step with ~0.09 errors (9/96 errors). There were important limitations, however, as we could not decompose the adipocytic tumors fully in step two, yet recognized MLS as distinct. Moreover, after compositing the spindle-like tumors PMS/LMS/DCS in step one, we could not decompose this artificial grouping. Accepting these limitations, the sum error over both steps was 0.14 (n = 9 errors in step one, and n = 4 errors in step two). Note that the samples wrongly classified in step two were classified correctly in step one thus having no carry-over error in this particular dataset. The types of error in step one are summarized in Table 2, and the error for each component of step two is shown in Table 3.

### Molecular fingerprints

**Figure 3:**
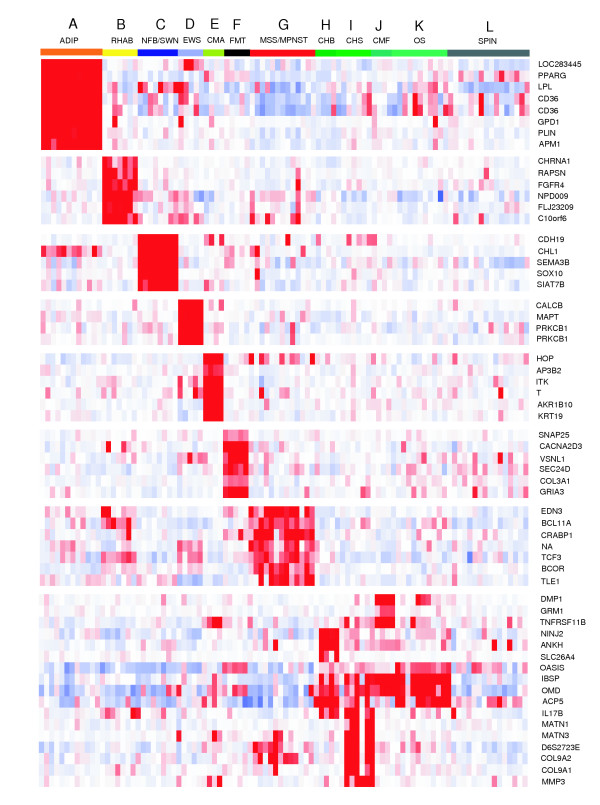
Pathognomonic fingerprints for many tumor types. In step one of our model, 61 genes were used in all folds of cross-validation. Average linkage clustering of this geneset reveals strong sets of distinct genes for many single mesenchymal tumors or composite groups. The sample types are color coded as before. A, adipocytic tumors; B, rhabdomyosarcoma; C, NFB/SWN; D, EWS; E, CMA; F, FMT; G, MSS/MPNST; H, CHB; I, CHS; J, CMF; K, OS; L, spindle-like tumors.

Feature selection of genes was carried out for each fold of our model, and while 237 distinct genes were encountered during cross-validation only 61 were selected in every fold (96 folds for leave-one-out cross-validation, see Materials and methods: feature selection). This robust 61-gene signature is illustrated in the gene-clustered heatmap of Figure 3. The adipocytic tumors (LMA/WLS/MLS), rhabdomyosarcoma, NFB/SWN, EWS, CMA, desmoid fibromatosis (FMT), and to a lesser extent MSS and MPNST have clear signatures. The CHS, chondromyxoid fibroma (CMF) and OS have less distinct patterns. However, the poorly differentiated spindle-like tumors have no distinct pattern. Yet this does not prevent our classification algorithm from recognizing the spindle-like tumor group as a whole. Negative expression of all other markers by the spindle-like tumors is informative to the k-NN algorithm just as it would be to a histopathologist. Additional data file 2 (sheet a) provides the gene information for Figure 3, including probeset identifications, full names, accession numbers and references describing their relation to respective pathways of differentiation and/or involvement in cancer.

**Figure 4:**
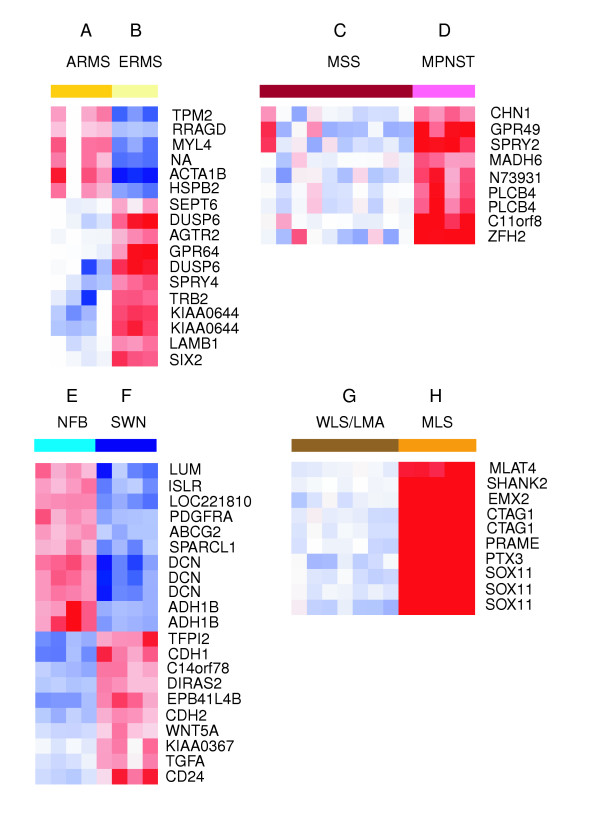
Pathognomonic fingerprints step two. Molecular fingerprints of genes for A and B: ARMS and ERMS; C and D: MSS and MPNST; E and F: NFB and SWN; G and H: WLS/LMA and MLS. We have selected genes based upon their inclusion in the majority of folds of cross-validation then clustered them by average linkage.

From the molecular fingerprint used in step two we selected genes used in the majority of cross-validation folds for display in Figure 4. Again these genes are fully described in Additional data file 2 (sheet b) online and some are discussed below.

## Discussion

We demonstrate here the feasibility of using GEM and machine learning to aid the diagnosis of mesenchymal tumors. In contrast to previous studies, our work encompasses a wide range of mesenchymal neoplasms and reflects the open-ended nature of histopathological diagnosis. It is clear from unsupervised methods such as MDS that there is information within the expression profile that can be further refined (Figure 1). This complex problem is surprisingly tractable firstly because of the large number of definitive gene markers associated with many of the tumors, especially the well-differentiated groups. In machine learning terminology we may state that these genes (or features) have no overlap in their class distributions, while in histopathological parlance their expression is pathognomonic. This would be rare in prognostic studies, for instance, as certain patients with good molecular signatures may still have poor outcomes due to unobserved factors. Secondly, we have used a novel machine learning strategy breaking this complex problem into soluble steps. We have implicitly admitted limitations in our model by not attempting to further decompose groups such as LMS/PMS/DCS (spindle-like tumors) and WLS/LMA. Thirdly, we have used a feature selection strategy that captures information evenly on all the tumor groups. Further validation on prospective samples is required before GEM studies are of clinical use for the diagnosis of mesenchymal tumors.

Cross-validation of the first step of our model gives 0.09 errors. This is a useful guide but its accuracy should not be overstated. A reliable estimate of error usually requires hundreds of samples per class, and a leave-one-out estimation is likely to underestimate error [30]. Moreover, there is the problem of statistically uncontrollable bias in our wide but shallowly sampled dataset [31]. However, our model is not grossly over-fitted (see Table 2) and more importantly the generalization to other datasets is clear from the biology of the molecular fingerprints (arising from feature selection). These fingerprints may be useful as the basis of a custom diagnostic chip and also provide insight into the etiology and cell of origin of certain tumors (see Additional data file 2 (sheets a and b online).

Several of the fingerprints are clearly related to the metabolism or function of differentiated mesenchymal tissues. For instance, adipocytic tumors are identifiable by their lipid-associated genes such as *perilipin (PLIN)*, *lipoprotein lipase (LPL)*, and *glycerol-3-phosphate dehydrogenase 1 (GPD1) *(Figure 3a). Similarly, rhabdomyosarcoma are characterized by genes such as *cholinergic receptor alpha (CHRNA1) *and *receptor-associated protein of the synapse (RAPSN) *associated with the musculoskeletal synaptic junction (see Figure 3b and Additional data file 2 (sheet a) online for supporting references).

When resolving these classes further in a second classification step some of the fingerprints reflect degrees of differentiation. Thus, the ARMS is defined by its higher expression of *tropomyosin-2 *(TPM2)*, skeletal muscle actin *(ACTA1) and *myosin-light polypeptide-4 *(MYL4), indicative of more maturity than the ERMS (see Figure 4a and 4b and supporting references in Additional data file 2 (sheet b) online).

Within these highly focused signatures there are clues to the origins of some of the poorly differentiated tumors. One striking similarity of gene expression is between the MPNST and the MSS (see Additional data file 1). The MSS have a simple karyotype consisting of an aberrant SYT-SSX fusion. The MPNST have a complex karyotype and are believed to arise from Schwann cell precursors (they are more malignant than SWN and frequently associated with NF1 mutations [32]) derived from the neural crest rather than the mesenchyme [12]. Both MPNST and MSS are aggressive and poorly differentiated. Their composite signature (Figure 3g) contains the gene *Endothelin-3 (EDN3)*, a key molecule in the development of the neural crest. *EDN3 *promotes self-renewal of multi-potent neural crest precursors or de-differentiation of matured cell types including Schwann cells [33-35].

Despite their similarity, the MPNST and MSS have distinct molecular signatures (Figure 4c and 4d) as do the NFB and SWN (Figure 4e and 4f), which are typically difficult to distinguish immunohistochemically. For instance, SWN but not NFB express the secreted glycoprotein *WNT5A *recently linked to metastases of OS [36] and melanoma [37]. Yet SWN is a benign tumor that never metastasizes.

Some genes could make useful monoclonal antibodies for immunohistochemistry. Genes such as the *myxoid liposarcoma-associated transcript-4 *(*MLAT4*), which as its name suggests distinguishes MLS from WLS/LMA (Figure 4g and 4h), appear to have been identified in molecular screens but not pursued as markers thus far. The overall expression of MLS is intriguing as it shows similarities to the putative neural crest tumors MPNST and MSS and the small round blue cell tumors ERMS, ARMS and EWS. Similarly, the fingerprint itself contains early mesenchymal and neural development genes (*EMX2*, *SOX11*), and neural restricted genes (*SHANK2*; a post-synaptic molecule). The profile also confirms previous reports of the expression of the immunotherapeutic targets *PRAME *and *CTAG-1 *(also known as *NY-ESO-1*) [38]. These are so-called 'germ cell antigens' because of their restriction to the testes of healthy males.

There is another clue to cellular origin within the CMA fingerprint. The overall expression pattern of CMA is closely related to the chondroblastic tumors CHS and chondroblastoma (CHB) (Figure 1). Likewise, the CMA are known to commonly contain focal regions of chondroid differentiation or more rarely chondrosarcomatous elements. As the tumor occurs solely along the midline it is proposed to originate from remnant notochord, an embryonic structure that is known to persist at least into infancy [39,40]. The expression of the *brachyury *(T) which is highly expressed in developing notochord strongly supports this theory [41,42].

Not all tumors had distinctive markers. The LMS, PMS and DCS were not distinguishable from each other. Cross-validation of our model in step one was fairly successful as our algorithm collectively recognized these tumors through a lack of specific markers, perhaps analogous to the way in which histopathologists recognize them (Figure 3l). At least part of the success of our model in step one is that it incorporated this uncertainty implicitly by compositing these tumors into a single 'spindle-like' group.

There are a number of extensions to the current study that may bring GEM technology closer to clinical use. Firstly, a larger study focusing on the poorly modeled cases (PMS/LMS/DCS) may help to elucidate categories supplementing current histological guidelines. Yet the pleomorphism and complex karyotypes of some mesenchymal tumors may confer a continuum of molecular pathology irreducible to simple categories. Such an analysis is unlikely to find adherents if it does not correspond with an improved interpretation of the histopathology or with appreciable clinical differences such as prognosis.

Secondly, by sampling all groups more thoroughly we could manage uncertainty. Our model gives a simple prediction of tumor type appropriate for the broad base and low sample numbers in our dataset. A more nuanced approach would be to calculate a probability of tumor type. This could be achieved most simply using Bayesian classifiers that implicitly calculate class membership probability. Thus in a clinical setting a sarcoma could be identified to a histopathologist as a central or classical example of its type, or be highlighted as an uncertain outlier for further immunohistochemical examination.

Thirdly, there is a large quantity of relevant molecular information that may transcend the diagnostic categories investigated here, yet may have prognostic significance. There is great interest, for instance, in both drug-resistance genes [43] and signatures associated with metastasis [44], which are common to a number of different tumor groups. Finally, a custom chip and user-friendly software that incorporates both a predictive model and such additional knowledge needs to be developed. This package might incorporate a prediction algorithm, visualization tools, plus additional housekeeping genes to aid normalization and quality controls. The molecular fingerprints shown here and fully listed in the additional data files (Figure 3 and 4 and Additional data file 2 online) could form the core of such a custom chip.

## Materials and methods

### Tumor specimens

Tumor biopsies were obtained from 96 participants presenting at the London Bone and Soft Tissue Tumour Service (Royal National Orthopaedic Hospital, Stanmore and University College London Hospitals, London), Great Ormond Street Hospital, London, or the Nuffield Orthopaedic Center, Headington, Oxford, in the UK. The diagnosis was determined by pathological examination using criteria established by the World Health Organization (WHO) tumor classification of soft tissue and bone tumors [45]. Where necessary, RT-PCR was performed to confirm common translocations (such as EWS, ARMS, MLS and synovial sarcoma). Ethical committee approval was obtained from all three treatment centers for the collection of fresh samples for this study. Clinical data such as diagnosis, site, grade and stage, age and sex are summarized in Additional data file 3 online. Patient biopsies were snap frozen with liquid nitrogen prior to RNA extraction and stored at -70°C.

### Nucleic acid extraction

Frozen sections from each tumor sample (needle core or resection) were examined microscopically prior to RNA extraction to confirm that the sample was representative and contained more than 80% tumor cells. Total RNA was extracted from adjacent tissue sections with >80% tumor cell content, using TRIzol™ reagent (Invitrogen Ltd, Paisley, UK) followed by purification with RNeasy columns (Qiagen Ltd, Sussex, UK) according to the manufacturer's instructions. RNA quality and quantity was assessed using RNA 6000 Nano chips on Agilent 2100 Bioanalyser according to manufacturer's instructions (Agilent Technologies UK Ltd, Cheshire, UK). Our RNA quality-control threshold for the rRNA peak ratio was 28s/18s ≤ 2.

### Microarray processing

The biotinylated hybridization target (biotin cRNA) was prepared from 10 μg of total RNA as previously described [46,47]. The quality and quantity of the biotinylated cRNA was checked prior to hybridization using the RNA 6000 Nano chips on Agilent 2100 Bioanalyser according to manufacturer's instructions. A total of 20 μg of the biotinylated probe was hybridized to Affymetrix HG-U133A Human GeneChips (Affymetrix^®^, Santa Clara, CA, USA) according to manufacturer's recommendations. In cases where smaller amounts of total RNA were obtained (EWS and rhabdomyosarcoma <5 μg total RNA), 150 ng of total RNA was subjected to two rounds of amplification to obtain a biotinylated cRNA yield of 20 μg according to Affymetrix recommendations (Genechip^® ^Eukaryotic small sample target labeling assay version II). Parallel hybridizations of probe synthesized from 10 μg and 150 ng (via single and double amplification respectively) for the same tumor sample were found to have similar quality-control and expression profiles (see ARMS, EWS samples in Figure 1). Hybridization of the synthesized biotinylated probes and scanning of the images were performed as previously reported according to Affymetrix recommendations [46,47].

### Data analysis

Data analysis was carried out using the R statistical environment and programming language [48]. We extensively used R software packages from Bioconductor [49], an open source bioinformatics resource. We used the 'affy' package written to handle Affymetrix data, and specifically the 'rma' algorithm for pre-processing, normalizing and calculation of expression values [50,51]. A modified version of the 'ipred' package was used for cross-validation and machine learning [52] together with the 'limma' package which was used for feature selection [53] (both described more fully below).

### Hierarchical clustering and MDS

The Cluster and Treeview software packages were used to produce the average linkage hierarchical clustering shown in Figure 3 and 4 (correlation distance) [54]. MDS was chosen for Figure 1 instead of hierarchical clustering as this captured the complexity of the data optimally within the space available. We used the classical MDS method (or principle coordinates method part of the R 'base' package) [48]. The stress metric measures distortion required to plot high-dimensional data [55].

### Classification algorithm

We chose simple k-NN pattern classification to model the data. This is a non-parametric algorithm, here based upon a table of inter-sample Euclidean distances [56]. The identity of an unknown (or test) sample is attributed by majority vote to that of the k = 3 nearest neighbors. We chose k = 3 due to the limiting minimum of three samples in our smallest class (DCS). The k-NN method was comparably effective to other methods tested (linear models, support vector machine, Bayes classifier; results not shown). We used leave-one-out cross-validation to estimate the error of our model. This method iteratively builds a predictive model from all combinations (or folds hereafter) of the data excepting one sample. The excepted sample is used to test the model error, the error being the proportion of inaccuracies from all folds. The multiple tumor classes and the limited number of samples in each class are unsuitable for accurate estimation of the classification error. This is therefore only a useful guide as the fingerprints are more important than the error. As a guide only, we used a permutation test to calculate the baseline error that might be expected from random guessing. Simply, this involves randomly permuting the class labels of the data ten times and counting the fraction of correctly corresponding labels with the original labels. To assess potential over-fitting we repeated this random permuting of the data class labels ten times but asked the computer to model the data and cross-validate, each time counting the fraction of errors. This does not, however, demonstrate a lack of over-fitting but merely a lack of gross over-fitting.

### Feature selection

As most expression data are uninformative, or even confounding of pathological categories, feature selection is incorporated into each fold of cross-validation. For multi-class models we used a well-suited feature selection method to capture information from each of the 19 tumor classes (not to split classes in step two). The algorithm selects 10 genes for each class that are different in expression from all others. We tested 5, 10, 20 and 30 gene sets finding 10 to be sufficient (better than 5 or 30 and as good as 20). We used the 'limma' method for each comparison [53]. Limma uses a variant of linear models with an empirically moderated estimate of the standard error effectively borrowing information from the ensemble variance of genes to aid inference about individual genes. This gives improved statistical power for small sample sizes.

## Additional data files

The following additional data are included with the online version of this article: an average linkage hierarchical clustering of the tumor samples using the same distance matrix as in Figure 1 (additional data file 1), a table providing the gene information for Figures 3 and 4 (additional data file 2) and a table containing clinical and pathological details of all samples used in this study (additional data file 3).

## Supplementary Material

Additional data file 1 Average hierarchical clustering of tumor samples. The same set of inter-sample distances were used as in Figure 1. The cophenetic correlation of this clustering, a measure of the summary quality, was 0.71. Click here for file

Additional data file 2 Supplementary information on the genes shown in Figures 3 and 4 in Worksheet S2A and S2B respectively. This includes references to the function of genes that lend support to our model feature selection, plus references the previously reported role of many genes in cancer. Click here for file

Additional data file 3 Clinical and pathological details of all samples used in this study. Click here for file

## Figures and Tables

**Table 1:** Errors of the simplest machine learning model

Model (n = 96)	Error +/- S.E.
Cross-validation	0.33
Random guessing (n = 10)	0.93 +/- 0.01
Re-permuted and cross-validated (n = 10)	0.94 +/- 0.01

We used leave-one-out cross-validation of all samples simultaneously. With random guessing we compared the matching of the true order of classification labels against a randomly generated set, which produces a baseline for cross-validation comparison. For re-permutation, the true labels were randomized to create a semi-random false dataset.

**Table 2:** Frequency table of errors

	*ADIP*	*CHB*	*CHS*	*CMA*	*CMF*	*EWS*	*FMT*	*MSS MPNST*	*NFB SWN*	*OS*	*RHAB*	*SPIN*
*ADIP*	12											
*CHB*		3										
*CHS*			5							1		2
*CMA*				4								
*CMF*					4							
*EWS*						5						
*FMT*							5					
*MSS MPNST*								11				
*NFB SWN*									8			
*OS*		1						1		9		
*RHAB*											7	
*SPIN*			2					1		1		13

The frequency table shows the agreement between the sarcoma classes and the cross-validation prediction along the diagonal. The true numbers of tumors in each group can be calculated by summing the numbers in columns and the predictions of our model from summing the rows. ADIP, adipocytic tumors; RHAB, rhabdomyosarcoma; SPIN, spindle-like tumors.

**Table 3:** Errors of two-step machine learning model

Step one	Error (absolute number)
All sarcoma (n = 96)	0.09 (9)

Step two	

MSS and MPNST (n = 14)	0.14 (2)
NFB and SWN (n = 8)	0.125 (1)
ARMS and ERMS (n = 7)	0.14 (1)
LMA/WLS and MLS (n = 12)	0

Cross-validation errors for step one and two of our prediction model are shown. The algorithm focuses on these samples of each row in isolation. Note that all the samples wrongly classified in step two were correctly classified in step one.
