# Supplementary figures and images for: A molecular map of mesenchymal tumors
Source: Genome Biol. 2005 Aug 26;6(9):R76. doi: 10.1186/gb-2005-6-9-r76 (PMC1242211; doi:10.1186/gb-2005-6-9-r76)

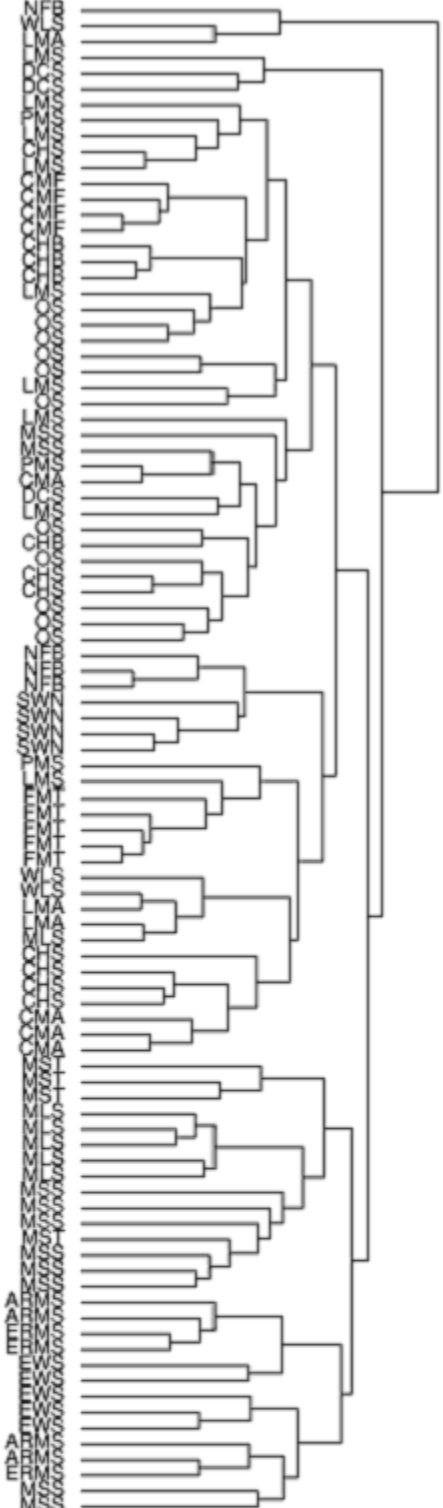

Supplement: Additional data file 1 — Average hierarchical clustering of tumor samples. The same set of inter-sample distances were used as in Figure 1. The cophenetic correlation of this clustering, a measure of the summary quality, was 0.71. [file gb-2005-6-9-r76-S1.pdf]
